# Supplementary material for: Accurate De Novo Prediction of Protein Contact Map by Ultra-Deep Learning Model
Source: PLoS Comput Biol. 2017 Jan 5;13(1):e1005324. doi: 10.1371/journal.pcbi.1005324 (PMC5249242; doi:10.1371/journal.pcbi.1005324)
Supplement: S1 Table — (DOCX) [file pcbi.1005324.s001.docx]

| 4qncA | 2mgyA | 4hyjA | 1kf6C | 2n6lA | 3emnX | 1p7bA | 1a0sP | 2lomA | 1mm4A |
| --- | --- | --- | --- | --- | --- | --- | --- | --- | --- |
| 3c02A | 2wscK | 1q90B | 3d31C | 2yevB | 4mbsA | 5a6eB | 5araT | 1oedC | 1orsC |
| 2wscL | 5ek0A | 2nq2A | 4wgvA | 4rngA | 4huqS | 4knfA | 4gycB | 2k0lA | 5c1mA |
| 3zevA | 4p6vB | 2ibzG | 2oarA | 1xioA | 4h33A | 5araW | 3fidA | 4ymkA | 4httA |
| 4he8E | 4rlcA | 4px7A | 3jcuW | 4pgrA | 2r6gG | 4ev6A | 5dl7A | 2wwbC | 4he8D |
| 3vmqA | 2llyA | 3dhwA | 2lnlA | 4d5bA | 4phzA | 4y28G | 2ge4A | 2yiuB | 2k21A |
| 1kqfC | 5i20A | 3rgwS | 4xnvA | 3oufA | 1c17M | 1m56B | 3effK | 4xk83 | 4zr1A |
| 3rbzA | 4k1cA | 2y69G | 4bpmA | 5a63C | 2lkgA | 4ymsC | 4bgnA | 3k3fA | 4cz8A |
| 5garO | 4u9lA | 4d6tD | 1yq3C | 4fqeA | 2x4mA | 3iyzA | 2y5yA | 2mm8A | 2lhfA |
| 4in5H | 2wscH | 3jbrE | 1ar1B | 5ekeA | 3tijA | 2gr8A | 1vf5D | 3jcuG | 3rkoA |
| 5ctgA | 5azbA | 5doqB | 2nmrA | 4x5mA | 3vwiA | 3tdoA | 2kyhA | 3b4rA | 4wd7A |
| 3jycA | 4l6rA | 4o9pA | 4ryiA | 4xxjA | 1occD | 1q16C | 4tquM | 1gzmA | 4czbA |
| 3rkoG | 4bemJ | 2wsc2 | 3vouA | 5hk1A | 4hycA | 4mqsA | 4quvA | 2mn6A | 2k73A |
| 2fynB | 3zjzA | 1occB | 2porA | 4kjrA | 4he8I | 5a63D | 5d0yA | 4cadC | 4xydB |
| 4dxwA | 2d57A | 3jcuD | 1t16A | 4qndA | 3rkoF | 3ddlA | 2m6bA | 4o6yA | 1xl4A |
| 3a2sX | 1mprA | 2mmuA | 1uunA | 1qleC | 2ervA | 2wscF | 3anzA | 4g1uA | 4rjwA |
| 1yq3D | 4g7vS | 2jafA | 4ltoA | 1rwtA | 4tq3A | 5f1cA | 3qe7A | 2lmeA | 3x29A |
| 5i32A | 2o01F | 4fuvA | 4od4A | 2j58A | 5dl5A | 3dl8E | 2kseA | 4rl8A | 2h8aA |
| 2o9dA | 2lckA | 1izlA | 4ky0A | 2ksfA | 5dirA | 1fw2A | 4g80I | 4gd3A | 3cn5A |
| 4chvA | 1vclA | 2lorA | 3vr8C | 2wsc3 | 2a9hA | 2m3gA | 4tquN | 5cfbA | 4ea3A |
| 2m8rA | 3p5nA | 4y7jA | 1p4tA | 3s0xA | 3wxvA | 3hw9A | 4frxA | 3b5dA | 5ivaB |
| 3x2rA | 3vr8D | 4m58A | 2zxeB | 2iubA | 3o7pA | 4f4lA | 3rlbA | 1tlwA | 2pnoA |
| 3iz1A | 3j1zP | 2ks9A | 5aymA | 2loqA | 4a2nB | 2w1pA | 2bl2A | 2mafA | 2oauA |
| 4j72A | 3bryA | 2q67A | 3ux4A | 4huqT | 4uc1A | 1qd6C | 1h6s1 | 4he8C | 3qnqA |
| 2ksdA | 1bccE | 3ukmA | 4l6v8 | 2wsc1 | 1uynX | 3kj6A | 1yc9A | 1nekD | 5ixmB |
| 1fx8A | 3qraA | 2a0lA | 4hw9A | 2bg9A | 5awwY | 3jcuX | 4p79A | 4in5L | 3dwwA |
| 3jcuR | 3um7A | 4o6mA | 4oo9A | 4he8A | 4p6vE | 4twkA | 5id3A | 5awzA | 5gaqA |
| 2ziyA | 3gi8C | 2kogA | 4dveA | 4o9pB | 4ytpD | 4qtnA | 4y25A | 2gfpA | 3l1lA |
| 1kf6D | 4z7fA | 3qq2A | 2j7aC | 4e1tA | 3mk7C | 4ezcA | 4ldsA | 2losA | 4rfsS |
| 3udcA | 3j9tR | 2evuA | 3sljA | 4n75A | 5doqA | 2lp1A | 3mk7B | 2qomA | 1vf5B |
| 2f93A | 4n74A | 4or2A | 5a1sA | 2f93B | 1fftC | 2f1cX | 2q7mA | 4b4aA | 3m71A |
| 4zr0A | 2z73A | 2m0qA | 5c8jI | 1yewC | 3emoA | 3tx3A | 1fftB | 4zp0A | 4f35A |
| 4l6v6 | 2n4xA | 4q2eA | 2f95B | 1oedB | 5bwkE | 4bwzA | 4o9uB | 5a43A | 4xu4A |
| 3x3bA | 2ksrA | 3chxB | 3v5sA | 1izlC | 2yiuA | 5gaeh | 4p6vD | 2xzbB | 4kt0F |
| 2vpwC | 4c9jA | 2b2fA | 3dwoX | 5a40A | 4njnA | 3kp9A | 4hzuS | 1k24A | 4d6uD |
| 5iofA | 1pw4A | 4kt0K | 4ri2A | 3a7kA | 3pjsK | 3wo7A | 3wmmM | 4atvA | 5ee7A |
| 3nymA | 3g67A | 4cskA | 4y28L | 4rl9A | 3pwhA | 4kppA | 3wdoA | 4y28K | 4tkrA |
| 1q90A | 2wscG | 3llqA | 3zuxA | 5dl8A | 4us3A | 2gr7A | 4hkrA | 3q7kA | 1ehkB |
| 3chxC | 1e54A | 3b9wA | 4pirA | 1nekC | 3dzmA | 1kqfB | 4ytpC | 1e7pC | 4ogqC |
| 4p6vF | 3ze3A | 2wjqA | 3jcuS | 2y69D | 4p6vC | 3ug9A | 4rdqA |  |  |
