# Supplementary material for: Accurate De Novo Prediction of Protein Contact Map by Ultra-Deep Learning Model
Source: PLoS Comput Biol. 2017 Jan 5;13(1):e1005324. doi: 10.1371/journal.pcbi.1005324 (PMC5249242; doi:10.1371/journal.pcbi.1005324)
Supplement: S2 Table — (DOCX) [file pcbi.1005324.s002.docx]

| 2n5uA | 4yo5L | 4z80D | 4z3uC | 4ufqB | 5c21B | 5cnqA | 4z3uD | 4z39B | 4x1tA |
| --- | --- | --- | --- | --- | --- | --- | --- | --- | --- |
| 5an6C | 4zi2C | 5ag8B | 4xvnF | 4yamD | 4zlhB | 5fbyA | 4xywA | 4yxyD | 4zuyB |
| 2n5dA | 2n24A | 4yy2D | 4zv0A | 2n52A | 2n8hA | 5byoB | 4zv0B | 2n5lA | 4zrsB |
| 2myhA | 4zv4D | 4y25A | 4zuaB | 4yo2A | 4xmqB | 2n11A | 2n4pA | 2n2cA | 3x27D |
| 4wy9A | 4v17B | 4rzaA | 4rhzB | 4xe7A | 4xzvH | 4xrwA | 4zi8B | 4xhpA | 4y68D |
| 4x0nB | 4zi9B | 2n12A | 4y6tF | 5awwG | 2n2eA | 4zwtN | 4v0hD | 5craB | 2n32A |
| 5bu3D | 5ekpD | 2n5nA | 4wbeC | 2mz0A | 5eo9B | 4ru3A | 2n8oA | 2mx7A | 4ru4F |
| 2n8pA | 4rocA | 4ru5C | 4yo3L | 4z80B | 4rs1B |  |  |  |  |
