# Supplementary material for: Accurate De Novo Prediction of Protein Contact Map by Ultra-Deep Learning Model
Source: PLoS Comput Biol. 2017 Jan 5;13(1):e1005324. doi: 10.1371/journal.pcbi.1005324 (PMC5249242; doi:10.1371/journal.pcbi.1005324)
Supplement: S3 Table — (DOCX) [file pcbi.1005324.s003.docx]

| 5emxB | 5evgA | 5gyqA | 5hy7C | 5idoA | 5ifsC | 2n86A | 2n8fA | 2ncgA | 5b1rA |
| --- | --- | --- | --- | --- | --- | --- | --- | --- | --- |
| 5c9oA | 5f5pH | 5hmqF | 5b64B | 5b86B | 5ck0A | 5ck1A | 5f5tD | 5amtB | 5amuB |
| 5b2gG | 5cylH | 5d8mA | 5e4bB | 5aozA | 5b46B | 5b48D | 5djeB | 2nd2A | 2nd3A |
| 5dcjA | 5dqsD | 5hjqA | 5ig8B | 5ig9H | 2nc8A | 5azyB | 2mzoA | 2nd4A | 5dijA |
| 5dlkD |  |  |  |  |  |  |  |  |  |
