# Supplementary figures and images for: Accurate De Novo Prediction of Protein Contact Map by Ultra-Deep Learning Model
Source: PLoS Comput Biol. 2017 Jan 5;13(1):e1005324. doi: 10.1371/journal.pcbi.1005324 (PMC5249242; doi:10.1371/journal.pcbi.1005324)

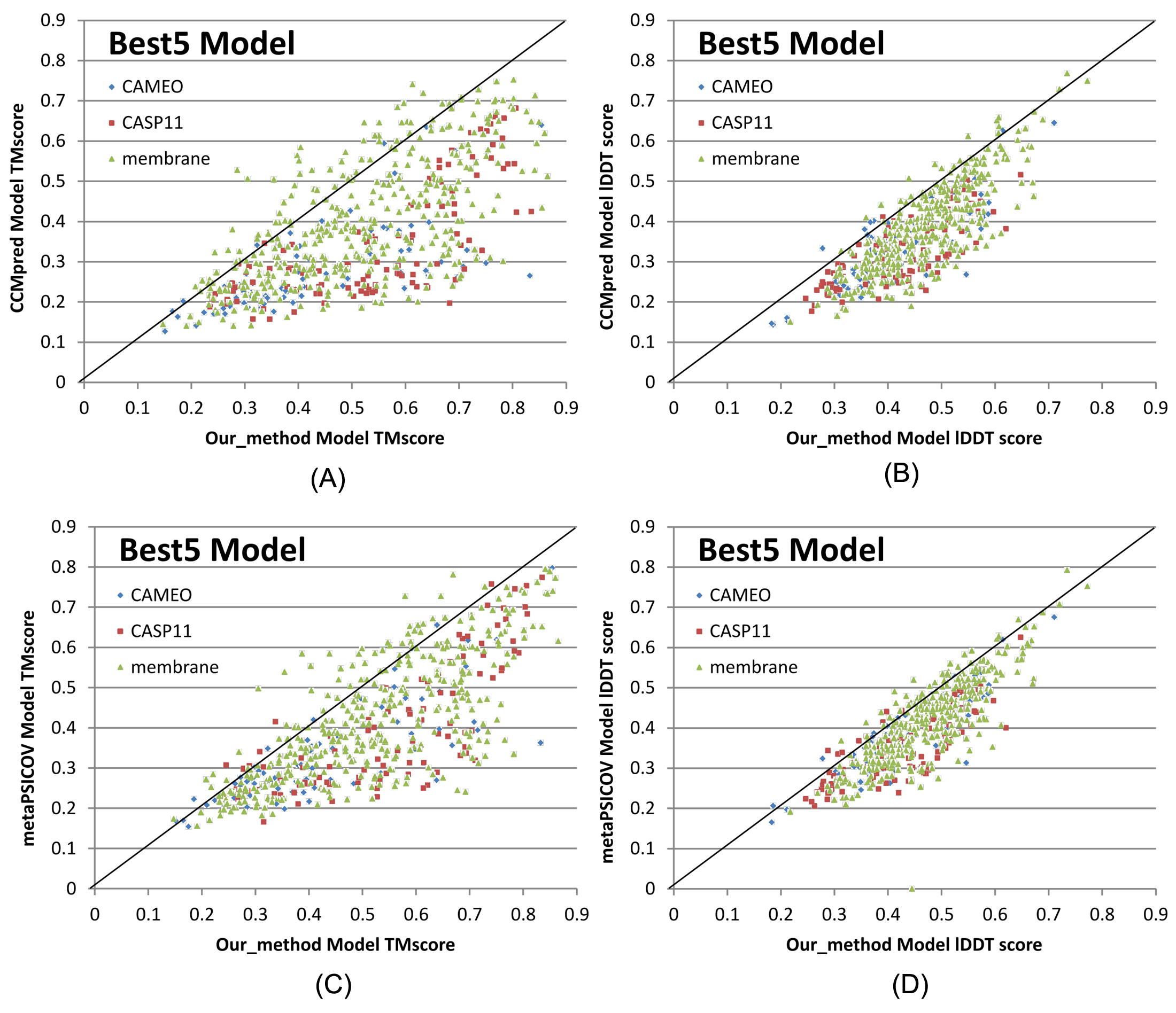

Supplement: S1 Fig — (A) and (B): comparison between our method (X-axis) and CCMpred (Y-axis) in terms of TMscore and lDDT, respectively. (C) and (D): comparison between our method (X-axis) and MetaPSICOV (Y-axis) in terms of TMscore and lDDT, respectively. lDDT is scaled to between 0 and 1. (TIF) [file pcbi.1005324.s004.tif]

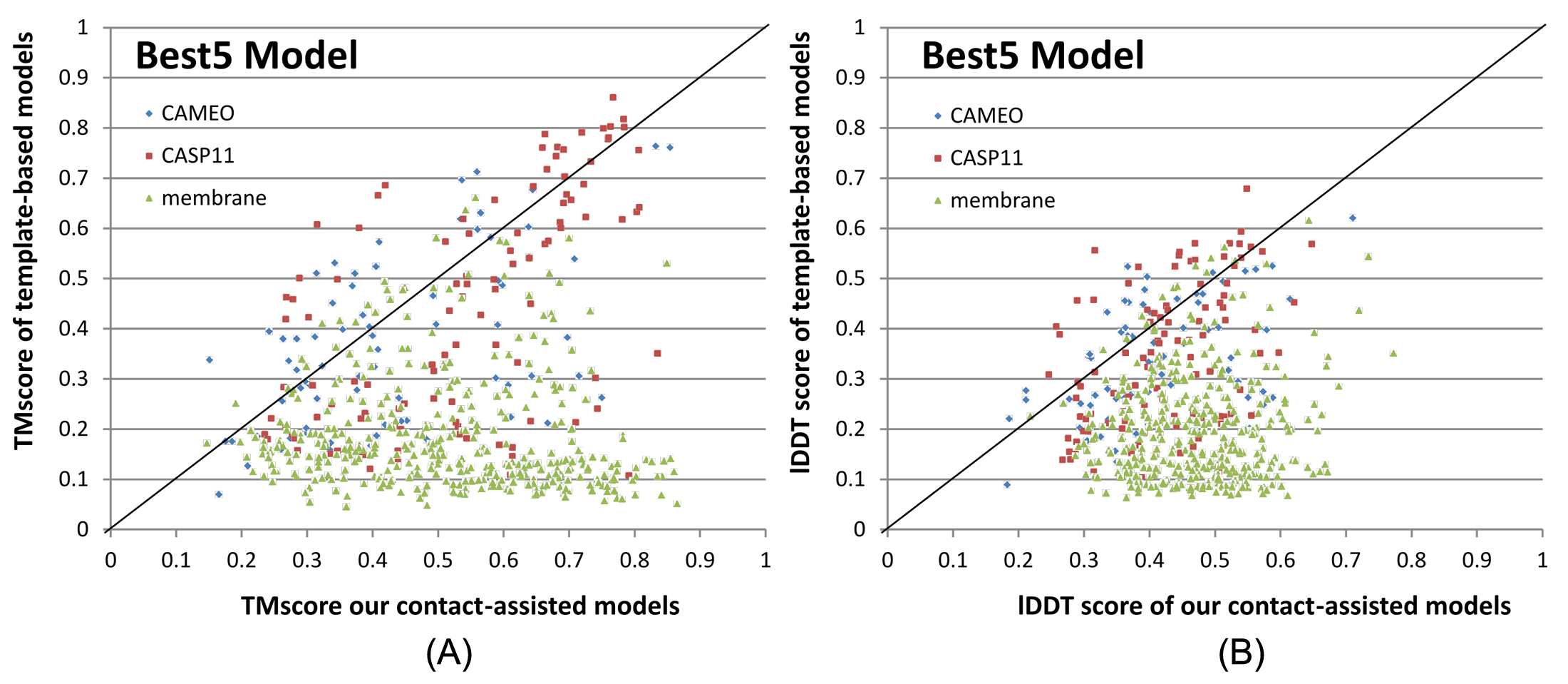

Supplement: S2 Fig — Comparison between our becontact-assisted models of the three test sets and their template-based models in terms of (A) TMscore and (B) lDDT. The best of top 5 models are evaluated. (TIF) [file pcbi.1005324.s005.tif]
